# Supplementary material for: Are carbon emissions trading and green financial instruments synergistic? -Comprehensive quantitative research based on content analysis
Source: PLoS One. 2024 Mar 7;19(3):e0298601. doi: 10.1371/journal.pone.0298601 (PMC10919601; doi:10.1371/journal.pone.0298601)
Supplement: S1 Data — (ZIP) [file pone.0298601.s001.zip › Data/地级市Z_F工作报告.zip!/┴╔─■/╔≥╤⌠╩╨/└┤╫╘╣1⁄2╓┌║┼í╛┬φ┐╦╩2╛▌═°í┐_2013.docx]

各位代表：

　　现在，我代表市政府向大会报告工作，请予审议，并请市政协各位委员提出意见和建议。

　　一、过去五年政府工作回顾

　　过去五年，在市委的正确领导下，在市人大及其常委会依法监督和市政协民主监督下，市政府团结带领全市人民，深入贯彻落实科学发展观，紧紧围绕完成“五大任务”、实现“三大目标”，抢抓机遇、开拓进取，圆满完成了市十四届人民代表大会确定的各项任务，沈阳老工业基地振兴取得了新成就。

　　——预计2012年全市地区生产总值实现6700亿元，比上年增长11.0%，比2007年翻一番；

　　——固定资产投资完成5600亿元，比上年增长22.8%，是2007年的2.8倍；

　　——社会消费品零售总额实现2798亿元，比上年增长15.3%，是2007年的2.3倍；

　　——公共财政预算收入完成715亿元，比上年增长15.3%，是2007年的2.5倍；

　　——城市居民人均可支配收入实现26430元，比上年增长13.3%，是2007年的1.8倍；农民人均纯收入实现13260元，比上年增长14.5%，是2007年的1.9倍；

　　——城镇登记失业率为3%，五年来始终保持在较低水平；

　　——单位地区生产总值能耗比2007年下降19.7%，化学需氧量、二氧化硫排放总量分别下降27.9%和15.3%，圆满完成省政府下达的各项节能减排任务。

　　过去五年，经济结构不断优化，综合实力持续提升。坚持以增量调整经济结构，以创新促进产业升级，经济发展的协调性进一步增强。

　　项目建设取得较大进展。五年开工建设投资100亿元以上项目34个，华晨宝马新工厂、上通北盛(三期)等一批质量高、效益好的重大项目已经投产或正在建设，为经济发展和结构调整提供了强大支撑，我市企业“山多峰少”的现状正在得到改变。

　　工业实力不断增强。大力实施工业“五项工程”，加快装备制造业三大聚集区和两大配套区建设，2012年规模以上工业总产值实现12850亿元，是2007年的2.7倍，工业增加值实现3200亿元，是2007年的2.3倍。汽车及零部件、机械装备、电子信息、现代建筑、农产品加工等5大产业实现千亿产出，28个重点产业集群销售收入实现6500亿元，其中19个超百亿元。华晨汽车、华晨宝马、上通北盛、机床、沈化、北方重工、远大、沈飞、沈煤、黎明航发、辽宁电力和东北电网等12家企业销售收入超百亿元，沈鼓20兆瓦电驱压缩机、沈变百万伏输变电设备等66个产品进入世界先进行列。

　　服务业发展全面提速。2012年服务业增加值实现2945亿元，比2007年翻一番；现代服务业占服务业比重为54.0%，我市成为国家现代服务业综合试点城市。东北总部基地等重大服务业项目加快建设，32个重点服务业集聚区营业收入突破3000亿元，其中7个超百亿元。金廊建设加速推进，成为高端业态集聚发展的现代服务业集中展示区。中街、太原街、长江街、兴工街等商业街区服务功能不断提升，华润万象城、恒隆广场、龙之梦亚太城等一批设施先进、功能完善的大型城市综合体投入运营。14家企业荣获“中华老字号”称号，五爱市场位居商品交易批发市场全国百强。东北区域金融中心初具规模，分行分公司以上金融机构已有118家。装备制造业物流集中发展区、临空现代物流港等物流园区和项目加快建设，全市营业收入超亿元物流企业已有38家。制博会、农博会、汽博会、陶博会、手机博览会等品牌展会知名度不断扩大，我市成为中国十大影响力会展城市。华强文化产业基地建设加快推进，和平区成为首批国家级文化和科技融合示范基地，全市文化产业增加值年均增长20.0%。沈阳国际旅游节影响力不断提升，2012年全市旅游总收入实现827亿元，是2007年的2.5倍。房地产市场始终保持平稳健康发展。

　　县域经济快速发展。2012年一市三县地区生产总值实现1273亿元，是2007年的3.6倍；公共财政预算收入完成88亿元，是2007年的6.2倍。农业基础设施建设不断加强，设施农业、高效特色农业面积快速增长，重要农产品实现有效供给，粮食产量达到81亿斤，实现“九连丰”。县域工业不断壮大，新民药业、辽中铸锻造机加、法库陶瓷、康平塑编等产业集群快速发展。县城建设步伐加快，城乡面貌变化明显。县域第一轮“三年倍增”计划提前完成，新民、辽中进入全国县域百强，法库进入全省县域第一集团。

　　新兴产业加快发展。大力培育和发展高端装备制造、电子信息、生物医药等战略性新兴产业，我市成为国家四大航空制造业基地之一，软件和信息服务业收入连续三年位居东北之首。国家创新型城市试点工作有序推进，产学研合作不断加强，新增国家级重点实验室8个、国家级质量检测中心9个，有46个项目列入国家重大科技专项。国际特种机床装备城成为国家级高新技术产业化基地，泗水科技城成为首批国家级科技成果转化示范基地。全市规模以上高新技术产品增加值完成1390亿元，是2007年的2.7倍。

　　过去五年，城乡面貌显著变化，城市功能不断完善。坚持全域规划、统筹发展，不断加快城乡建设步伐，2012年城建投资完成506亿元，五年累计投资1983亿元，年均增长18.5%。

　　空间布局进一步优化。全面修编城市总体规划，实施行政区划调整，东西南北中各具特色的空间格局基本形成。浑南新城建设日新月异，蒲河生态经济带建设全面启动，新城、新市镇建设步伐加快，城乡一体化发展全面推进。

　　基础设施建设成效明显。东一环、东二环快速路一期工程竣工通车，三环高速路改扩建工程部分通车，四环快速路全线贯通，五年新建改造公路3125公里，新建大中型桥梁92座，地铁1号线、2号线通车运营，地铁9号线、10号线完成前期工作，桃仙国际机场T3航站楼主体结构封顶，沈阳南站建设和沈阳站、沈阳北站改造进展顺利，沈本产业大道、沈彰开发大道、沈阜开发大道、沈抚二号公路建成通车，哈大高铁开通运营，连接城乡、辐射周边、便捷快速的立体化综合交通网络正在形成。全运会40个体育场馆新建改造工程全部完工，国际展览中心、城市规划展示馆等一批公共服务设施投入使用。大伙房水库输水工程竣工送水，西气东输工程向沈阳供气，智能电网加快建设，城市运行保障能力显著增强。

　　生态环境不断改善。大力实施城乡绿化，辽河、浑河、蒲河、棋盘山、卧龙湖等重点区域生态保护治理成效明显。完成9家热电厂及140余台大型燃煤锅炉脱硫改造，城市环境空气质量优良天数稳定在330天左右。新建污水处理厂24座、垃圾填埋场12个，城市污水处理率和垃圾集中处理率分别达到85.5%和100%。全国环境建设样板城创建工作扎实推进，9个涉农区县(市)、开发区全部通过国家生态区县验收，生态市创建工作全面达标。

　　城市管理水平不断提升。构建以市级为主导、区级为主体、街道为基础、社区为前沿的四级城管格局，推动城市管理重心下移。大力推进小街小巷和城中村、城边村改造，实施街路景观提升工程，加大拆违治乱力度，园林绿化档次、环境卫生质量和市容景观形象不断提升。改善环卫作业装备，道路机械化清扫率大幅提升。加大交通设施投入力度，新增更新公交车辆3700余台。全面开展“数字沈阳”建设，市区两级数字化城管平台投入使用，城市管理的精细化、信息化水平得到提升。

　　过去五年，改革开放全面推进，发展活力不断增强。深入推进改革开放，努力破解体制机制障碍，不断为老工业基地振兴注入新的活力。

　　重点领域改革不断深化。全面启动国家新型工业化综合配套改革，信息化与工业化融合、优化金融生态、统筹城乡发展和新型城镇化、文化体制、行政管理体制等重点领域和关键环节改革取得新进展。国有企业改革继续深化，公共资源交易平台建设扎实推进，土地集中统一管理进一步加强，行政审批事项始终保持全国副省级城市最少。农村改革不断深化，发展活力进一步增强。非公有制经济加快发展，经济总量占全市比重为65.9%。

　　对外开放水平稳步提升。五年实际利用外商直接投资277亿美元，26户世界500强在我市新设立企业44家。全市外贸进出口总额实现441亿美元，年均增长13.4%。新批境外投资项目141个，协议投资额7亿美元。沈阳综合保税区通过国家验收，法国、德国总领事馆开馆，韩国周、东北亚论坛成为对外交往的重要平台。加大对内招商力度，调入内资实现4652亿元。

　　沈阳经济区一体化建设步伐加快。8城市共同签署了系列合作发展框架协议，沈抚同城化等区域合作扎实推进，沈、抚、铁三市实现024电信区号共享，19个新城新市镇建设步伐加快，中心城市聚集辐射作用进一步增强。

　　过去五年，和谐社会建设成效显著，人民生活不断改善。坚持把保障和改善民生作为政府工作的根本出发点和落脚点，五年累计民生投入1266亿元，发展成果更多惠及全市人民。

　　就业和社会保障工作扎实推进。加大就业、创业扶持力度，每年约有10万名高校毕业生实现就业，困难家庭大学毕业生就业率达100%。实行困难群体就业援助，五年开发公益性岗位11000个，零就业家庭始终保持动态为零。全市企业普遍建立工资集体协商制度，合理调整初次分配，劳动关系更加和谐。全社会养老保险待遇水平逐年提高，城镇居民医疗保险、新型农村合作医疗年人均补助标准大幅提升。城乡社会救助体系不断健全，低保标准五年翻了一番。加大市区两级养老服务中心和社区日间照料站建设力度，全市养老机构新增床位1万张。我市成为全国实施妇女儿童发展纲要先进市和全国残疾人工作示范市。通过廉租房、公租房、经济适用房等保障方式，解决了20余万户低收入家庭住房困难。投入15亿元，改造弃管住宅小区1502个。投入4亿元，改造城市供水二次加压泵站612处、供水管网355公里。投入9.4亿元，解决了农村104.6万人口饮水安全。

　　社会事业全面发展。努力促进教育公平，提前两年完成“双高普九”任务，投资35.3亿元，新建改造107所九年一贯制学校、100所义务教育学校、216所农民工子女学校和5所万人规模职业学校，我市成为国家装备制造业职业教育试验区。各项医改工作全面推进，看病难、看病贵问题得到缓解。投资24.1亿元，实施7所市属重点医院改扩建和一市三县中心医院改造，就医条件得到改善。大力推进市艺术中心、工业博物馆等公共文化设施建设，加强文物遗迹修缮保护，广泛开展艺术惠民工程，艺术精品创作屡获大奖，我市连续四年成为全国文化体制改革先进地区。改造123个老旧社区公共用房，便民服务条件得到改善。城乡新建改造健身广场108个，增设户外健身器材4.1万件，市民拥有了更多的健身设施和休闲空间。

　　社会管理水平不断提升。加强法治政府建设，自觉接受市人大及其常委会的依法监督和市政协的民主监督，人大代表建议、政协提案办复率始终保持100%。五年提请市人大常委会审议地方性法规草案23件，发布实施政府规章36件。加强和创新社会管理，“党委领导、政府负责、社会协同、公众参与”的工作格局不断完善。新建改造68个公安派出所，加强治安防控管理体系建设，“平安沈阳”建设成效明显，社会治安状况持续好转。信访“沈阳模式”全国推广，安全生产形势保持稳定，食品药品监管力度不断加大，文明城市创建工作全面推进，城市文明程度和市民文明素质持续提升。

　　国防动员、人防和民兵预备役建设进一步加强，双拥模范城创建实现“七连冠”。民族团结进步事业不断发展，对口支援工作成效显著。国家安全、保密、统计、审计、工商行政管理、参事、宗教、侨务、贸促、广播电视、新闻出版、气象、防震减灾、档案、修志、文史、人口与计划生育、红十字会和慈善等工作都取得了新成绩。

　　各位代表，过去五年所取得的成就来之不易。这是市委正确领导的结果，是全市人民团结一心、努力拼搏的结果。过去五年，勤劳智慧的沈阳人民用激情和汗水谱写了和衷共济、开拓奋进的新篇章。在此，我代表市政府，向全市各族人民致以崇高的敬意！向人大代表、政协委员，向各民主党派、工商联、无党派人士和各人民团体，向驻沈人民解放军、武警部队官兵和中、省直单位，向所有关心、支持沈阳的港澳台同胞、海外侨胞和国际友人表示衷心感谢！

　　在总结成绩的同时，我们也清醒地看到，全市经济社会发展还面临着一些突出的矛盾和问题。一是经济结构调整力度还不够大，自主创新能力有待进一步增强；二是中心城市建设步伐还不够快，辐射带动能力有待进一步提升；三是一些关系人民群众切身利益的问题还没有解决到位，保障改善民生任务依然繁重。对此，我们一定要在今后工作中采取有力措施，进一步加以解决。

　　二、今后五年政府工作的主要目标和任务

　　今后五年，是沈阳实现老工业基地全面振兴和率先全面建成小康社会的关键时期。我们必须紧紧抓住国家继续深入实施振兴东北地区等老工业基地战略、支持沈阳经济区开展新型工业化综合配套改革的难得机遇，发挥比较优势，应对各种挑战，凝心聚力，攻坚克难，推动沈阳在科学发展道路上加速前行。

　　今后五年经济社会发展的奋斗目标是：全面完成“十二五”规划任务，努力实现“十三五”良好开局，到二○一七年，地区生产总值和城乡居民人均收入比二○一○年翻一番，基本实现老工业基地全面振兴，提前全面建成小康社会。

　　今后五年经济社会发展的主要任务是：

　　——深化改革开放，全面激发发展活力。深入推进国家新型工业化综合配套改革，务求在形成产业创新机制、完善科技创新体系、促进区域一体化发展等重点领域和关键环节取得重大突破，协同推进投融资管理、城乡统筹、社会管理、土地科学合理利用等各方面改革创新，着力破除制约经济社会发展的瓶颈和障碍。实施更加积极主动的开放战略，实际利用外商直接投资稳步增长，外贸进出口总额年均增长10%以上。建立健全区域合作机制，加快沈阳经济区一体化进程。

　　——推动转型发展，不断增强综合实力。把推进经济结构调整作为加快转变经济发展方式的主攻方向，不断提高经济增长的质量和效益。深入推进两化融合，改造提升传统产业，加快发展优势产业，大力发展战略性新兴产业，促进装备制造业走向智能化、成套化、高端化，全市规模以上工业总产值突破25000亿元，基本建成具有国际竞争力的先进装备制造业基地。扎实推进国家现代服务业综合试点，推动服务业走向高端化，现代服务业占服务业比重达到62%，加快建设东北区域金融中心、物流中心和现代商贸中心。加大城乡统筹力度，推动城乡一体化发展，一市三县地区生产总值和公共财政预算收入占全市比重分别达到25%和20%。实施创新驱动，加强人才培养和智力资源开发，着力构建完善技术创新和知识创新体系，全社会研发投入占地区生产总值比重达到3.5%以上，综合创新能力进入国家创新型城市前列。完善城乡规划体系，加强基础设施建设，提升城市功能，加快建设国家中心城市。

　　——大力发展文化事业和文化产业，加快建设文化强市。注重社会效益与经济效益的有机统一，推动文化事业全面繁荣、文化产业快速发展。深化文化体制改革，加强公共文化设施建设，完善公共文化服务体系，繁荣艺术精品创作，推进文化惠民工程，不断满足人民群众日益增长的多层次、多样化文化需求。加快重大文化产业项目建设，大力发展新型文化业态，文化产业增加值年均增长20%以上。加强历史文化遗产的保护和利用，挖掘沈阳文化底蕴，叫响历史文化名城品牌。大力加强社会公德和职业道德教育，弘扬中华传统美德和时代新风，提升城市文明和市民素质，建设国家文明城市。

　　——着力保障改善民生，努力构建和谐社会。把解决好人民群众最关心最直接最现实的利益问题作为保障改善民生的重中之重，努力让全市人民过上更好生活。实施更加积极的就业政策，推动实现更高质量的就业。千方百计增加居民收入，确保城乡居民收入与经济发展同步增长。继续扩大社会保险覆盖面，稳步提高社会保障水平，全面建成全覆盖、保基本、多层次、可持续的城乡社会保障体系。积极应对人口老龄化，加快发展老龄事业。全面实施素质教育，完善终身教育体系，促进教育均衡协调优质发展，努力办好人民满意的教育。深入推进医药卫生体制改革，为城乡群众提供安全有效方便价廉的公共卫生和基本医疗服务。加强和创新社会管理，进一步提高社会管理科学化水平。

　　——加强生态文明建设，打造生态宜居之都。把生态文明建设放在突出地位，推进绿色发展、循环发展、低碳发展，不断增强经济社会可持续发展能力，努力打造全国环境建设样板城。严格执行城市总体规划，推动各地区按照功能定位建设发展，形成科学合理的生产空间、生活空间和生态空间。推进节能减排，支持节能低碳产业发展，增强市民环保意识，提升全社会生态文明水平。继续实施“青山”、“碧水”、“蓝天”工程，城市环境空气质量稳步提升，努力把沈阳建设成为生态宜居的美丽家园。

　　三、2013年政府主要工作

　　今年是全面贯彻落实党的十八大精神的第一年，是新一届政府的开局之年，也是沈阳的全运之年，做好今年各项工作意义重大。今年政府工作的总体要求是：全面贯彻落实党的十八大精神，按照市委十二届五次全会部署，坚持以科学发展观为指导，围绕实现“三大目标”，扎实推进“五大任务”，突出做好稳增长、办全运、惠民生等重点工作，继续保持经济社会持续健康发展，实现新一届政府工作扎实开局。

　　今年全市经济社会发展的主要预期目标是：地区生产总值增长10%，固定资产投资增长15%，公共财政预算收入增长12%，社会消费品零售总额增长15%，城市居民人均可支配收入增长11%，农民人均纯收入增长12%，城镇登记失业率控制在4%以内。

　　今年要重点做好以下五方面工作。

　　(一)推动经济持续健康发展

　　把稳增长作为经济工作的首要任务，牢牢把握稳中求进的总基调，积极应对形势变化，强化经济运行组织协调，进一步增强经济发展的稳定性、协调性和可持续性。

　　加大投资工作力度。充分发挥投资对经济增长的拉动作用，突出抓好以290个市级重点项目为代表的一批重大产业项目，强化土地、资金等各类要素保障。继续开展“双进双解”活动，及时解决项目建设中遇到的困难和问题，确保项目按既定时间开工建设、按工期计划竣工投产。

　　全力推进工业发展。全面推进工业“五项工程”，坚持以龙头企业为核心，实施创新驱动，深化两化融合，不断提升工业竞争力，规模以上工业增加值增长12%。继续推动汽车及零部件、机床及功能部件、电力电气、通用及专用机械、现代建筑、农产品加工、医药化工、手机等优势产业加快发展，密切关注企业运行状况，支持优势骨干企业开拓市场、加快发展，加大对中小微企业扶持力度，帮助企业排忧解难、减轻负担。加快三大聚集区、两大配套区和特色产业园区建设，推动装备制造业不断提高自主创新能力、重大装备成套能力、基础产业配套能力和生产性服务业支撑能力。以现有优势产业为依托，大力发展IC装备、智能装备、通用航空等高端装备制造业及电子信息、生物医药、新能源汽车等战略性新兴产业，着力培育具有自主核心技术的领军企业，积极抢占新一轮发展制高点。抓好产业集群建设，加快华晨宝马发动机、上通北盛(三期)等重大项目及大东汽车城、铁西滨河新城、蒲河新城、现代建筑产业园和金属新材料产业园等重点园区建设步伐，吸引配套企业加速集聚。

　　加快发展服务业。深入推进国家现代服务业综合试点，推动服务业特别是现代服务业发展壮大。加快发展生产性服务业，推动生产性服务业与制造业分立、互动和融合发展。依托金融商贸开发区和金融街，加快金融机构集聚。推进沈阳国际物流港等大型物流园区、重大物流项目及浑南国家电子商务示范基地建设。大力发展生活性服务业，突出发展特色、打造知名品牌、推动产业集聚、培育领军企业。加快金廊建设步伐，打造国家级中央商务区。加快太原街、中街、南塔、五爱市场、兴华街、长江街、东中街、奥体商圈等商业街区的建设及业态升级，培育新兴消费热点，大力发展夜间经济，满足城乡居民消费需求。加快和平区国家级文化和科技融合示范基地、棋盘山国家文化产业示范区建设，推进皇城、北市等地区综合改造。精心办好品牌展会。依托历史文化、民俗风情和自然资源，大力发展特色旅游。认真落实国家调控政策，继续保持房地产市场平稳健康发展。

　　进一步增强县域经济实力。落实强农惠农政策，加大农田水利等基础设施投入，巩固农业基础地位，保障粮食和主要农产品有效供给。大力发展现代农业，开工建设100个规模以上农业产业化项目，加快蒲河生态经济带、沈康高速现代农业示范带和光辉现代农业示范区建设。充分发挥比较优势，做大做强优势产业，不断壮大产业集群，带动县域工业加快发展。继续推进“万村千乡市场工程”和“新网工程”建设，进一步完善农村流通体系。积极稳妥推进城镇化，加快县城和新城新市镇建设，努力增强城镇综合承载能力，有序推进农业转移人口市民化。

　　(二)提高城乡环境建设管理水平

　　紧紧抓住筹办全运会有利契机，推进基础设施、城乡环境和城市文明再上新台阶。

　　加大基础设施建设力度。启动一环、二环快速路二期工程，完成三环高速路改扩建和四环快速路新建工程，建设各环线之间连接线，改造新建一批主干道和桥梁。开工建设地铁9号线、10号线，推进沈阳至新民、沈阳至辽中轨道交通建设前期工作，浑南现代有轨电车投入运营。完成桃仙国际机场T3航站楼、沈阳北站、沈阳南站等交通枢纽改造建设。加快给排水设施和地下管网综合改造步伐。

　　着力改善生态环境。加快实施三四环沿线、机场路、沈西北防风阻沙带等重点区域绿化，完成植树造林40万亩。加强辽河、浑河、蒲河流域生态治理和棋盘山、卧龙湖生态保护利用，实施百里环城水系游园景观提升工程，完成浑河核心段滩地公园改造。加快推进雨污分流及西部污水处理厂扩建工程，南部污水处理厂投入使用。实施污泥无害化处理，推进生活垃圾分类收集和焚烧处理，完成农村环境连片整治示范工程。深入推进节能减排，实施燃煤锅炉脱硝改造，全市单位地区生产总值能耗下降3.77%，化学需氧量、二氧化硫排放总量分别下降2.5%和1.5%。确保通过国家环保模范城复检，力争生态市建设通过国家技术核查。

　　提升城市管理水平。强化城市精细化管理，加快“数字沈阳”建设，深入推进数字城管、三网融合等项工作。优先发展公共交通，加快停车场、过街通道等基础设施建设。加强机场、车站、城市出口路、全运场馆周边及旅游景区等窗口、区域和重点街路环境整治，提升主要街路亮化水平。

　　做好全运会服务保障。加速浑南新城建设步伐，完成“三村三中心”和各类体育场馆建设。精心做好竞赛组织、指挥通讯、来宾接待、医疗卫生、对外宣传、志愿者服务等工作，协助做好火炬传递、开闭幕式等大型活动，加强交通保障、食品安全保障和安全保卫。深入开展“迎接全运会、创建文明城”系列活动，努力形成全民参与全运、合力推动创城的良好局面。

　　(三)深入推进改革开放

　　大力推进新型工业化综合配套改革，进一步扩大对外开放，不断为经济社会发展提供新的动力与活力。

　　深化重点领域改革。加速推进信息化与工业化深度融合，加快市级试验区和示范企业建设，深化信息技术在关键环节的集成应用，力争在设计研发智能化、生产过程自动化、经营管理信息化等方面取得突破。深入推进科技体制改革，以企业为主体，以市场为导向，加强产学研合作，以创新驱动产业升级。建立健全科技创新指标体系，加快完善科技创新评价标准和激励机制，加快建设国家创新型城市。深化国有企业改革，推动重点国有企业股份制改造和战略重组，进一步释放国有企业发展潜能。以沈抚同城化和城际连接带新城新市镇建设为重点，加快沈阳经济区一体化进程。

　　统筹推进各项配套改革。深入开展金融创新，积极推进国家优化金融生态综合试验，大力发展产业金融，培育多层次资本市场。积极探索节约集约利用土地新机制和新模式，进一步提高土地利用率。总结于洪区、苏家屯区统筹城乡发展和新型城镇化试点经验，着力在城乡规划、基础设施、公共服务等方面加速城乡一体化发展步伐。总结东陵区、沈北新区行政管理体制改革经验，推动政府职能向创造良好发展环境、提供优质公共服务、维护社会公平正义转变。继续推进事业单位分类改革。

　　进一步提高对外开放水平。加大招商引资力度，鼓励支持大企业(集团)在沈设立总部(区域总部)和研发中心，实现利用外资和吸引内资稳定增长。推进出口基地建设，支持企业开拓海外市场。

　　(四)扎实做好民生工作

　　进一步加大民生投入，让全市人民共享振兴发展成果。

　　加强就业和社会保障工作。重点做好高校毕业生、农村转移劳动力、就业困难人员就业服务。强化政府、工会、企业组织三方协调机制，全面落实工资集体协商制度，积极构建和谐劳动关系。深化收入分配制度改革，千方百计增加居民收入。健全社会保障体系，不断提高社会保险待遇水平和城乡低保标准。推进养老服务设施建设，养老机构新增床位6000张，加快残疾人区域康复中心建设，加强农村困难家庭常年病人集中康复托管服务。

　　改善城乡居民生活条件。进一步解决城乡低收入家庭住房困难，完成保障性住房3.5万套，补贴农村贫困户建房3622套。继续实施“节能暖房”工程，推进弃管小区的有效管理。统筹推进菜市场、早餐店、便利店等网点布局，打造8-10分钟便民商圈，新建改造76个便民菜市场。推进城市供水管网改造，加强农村饮水安全工程建设和管理。继续实施小街小巷整修工程，推进街路无障碍设施建设，修建500公里农村公路，新增更新公交车辆1000台，新开调整公交线路20条，让市民出行更便捷、乘坐更舒适。

　　加快发展社会事业。深入推进教育均衡发展，继续实施百所义务教育学校提升工程，改造14所特殊教育学校，新建改造50所普惠制幼儿园和1所特殊教育幼儿园，加强教师队伍建设，提高师德水平和业务能力，加快职业教育国家试验区建设步伐。深化医药卫生体制改革，健全农村三级医疗卫生服务网络和城市社区卫生服务体系，推进县级公立医院改革，加快市一院(二期)、儿童医院(二期)和公共卫生服务中心建设。加快数字化图书馆、艺术中心等公共文化服务设施建设，继续实施艺术惠民工程。新建改造沿河健身带和一批农村社区文体广场，逐步实施学校文体设施向市民开放，市区两级全民健身中心全部投入使用。

　　加强和创新社会管理。改进政府服务方式，加强基层社会管理和服务体系建设，增强城乡社区服务功能。畅通和规范群众诉求表达、利益协调、权益保障渠道，加强重大决策社会稳定风险评估，强化安全生产和食品药品监管，严防各类重特大安全事故发生，严打食品药品领域违法犯罪行为，健全社会治安防控体系，依法防范和惩治各类违法犯罪活动，保障人民生命财产安全。继续深化双拥工作，加强国防后备力量建设，推进军民融合发展。

　　(五)加强政府自身建设

　　站在新的起点上推动全市经济社会实现持续健康发展，人民高度期盼，政府责任重大，必须以改革创新的精神全面加强政府自身建设，不断提高推动科学发展的能力与水平。

　　必须严格坚持依法行政。加强政府立法工作，强化行政执法监督，加大政务公开力度，努力建设法治政府。各级政府必须更加自觉地接受人大及其常委会的依法监督和政协的民主监督，坚持向人大报告工作和向政协通报工作，认真办理人大代表建议、议案和政协提案，不断提高依法行政水平。

　　必须增强公信力和执行力。建立健全科学民主决策机制，完善重大公共政策群众参与制度，确保各项决策体现民意、符合实际。加强软环境建设，创新行政管理方式，规范行政审批行为，提高政府行政效力和办事效率。各级政府一经做出决定决策，必须一抓到底、抓出成效，以实际行动兑现承诺、取信于民。各级领导干部要恪尽职守、率先垂范，进一步改进工作作风，把心思用在干事业上，把精力用在谋发展上，把本事用在抓落实上，直面矛盾、敢于担当、顾全大局、真抓实干，以奋发有为、昂扬向上的精神状态开创各项工作的新局面。

　　必须加强廉政建设。建立健全惩治和预防腐败体系，增强制度约束力，努力从源头上防治腐败。加强对政府重大投资项目、重大资金支出的监管和跟踪审计，严厉查处违纪违法案件，确保行政权力在阳光下运行。政府各级领导干部和全体公务员要克己奉公、廉洁自律、勤勉尽责，做人民满意的公仆。

　　各位代表，沈阳已经步入了科学发展的新阶段，踏上了全面振兴的新征程。让我们在市委的正确领导下，锐意进取，埋头苦干，为加速沈阳老工业基地全面振兴、率先全面建成小康社会而努力奋斗！
